# Pulmonary Tuberculosis and Delay in Anti-Tuberculous Treatment Are Important Risk Factors for Chronic Obstructive Pulmonary Disease

**DOI:** 10.1371/journal.pone.0037978

**Published:** 2012-05-25

**Authors:** Chih-Hsin Lee, Ming-Chia Lee, Hsien-Ho Lin, Chin-Chung Shu, Jann-Yuan Wang, Li-Na Lee, Kun-Mao Chao

**Affiliations:** 1 Graduate Institute of Biomedical Electronics and Bioinformatics, National Taiwan University, Taipei, Taiwan; 2 Department of Internal Medicine, Buddhist Tzu Chi General Hospital, Taipei Branch, New Taipei, Taiwan; 3 School of Medicine, Tzu Chi University, Hualien, Taiwan; 4 Department of Pharmacy, Buddhist Tzu Chi General Hospital, Taipei Branch, New Taipei, Taiwan; 5 Graduate Institute of Epidemiology and Preventive Medicine, National Taiwan University, Taipei, Taiwan; 6 Department of Traumatology, National Taiwan University Hospital, Taipei, Taiwan; 7 Department of Internal Medicine, National Taiwan University Hospital, Taipei, Taiwan; 8 Department of Laboratory Medicine, National Taiwan University Hospital, Taipei, Taiwan; McGill University, Canada

## Abstract

**Objective:**

Tuberculosis (TB) remains the leading cause of death among infectious diseases worldwide. It has been suggested as an important risk factor of chronic obstructive pulmonary disease (COPD), which is also a major cause of morbidity and mortality. This study investigated the impact of pulmonary TB and anti-TB treatment on the risk of developing COPD.

**Design, Setting, and Participants:**

This cohort study used the National Health Insurance Database of Taiwan, particularly the Longitudinal Health Insurance Database 2005 to obtain 3,176 pulmonary TB cases and 15,880 control subjects matched in age, sex, and timing of entering the database.

**Main Outcome Measures:**

Hazard ratios of potential risk factors of COPD, especially pulmonary TB and anti-TB treatment.

**Results:**

The mean age of pulmonary TB cases was 51.9±19.2. The interval between the initial study date and commencement of anti-TB treatment (delay in anti-TB treatment) was 75.8±65.4 days. Independent risk factors for developing COPD were age, male, low income, and history of pulmonary TB (hazard ratio 2.054 [1.768–2.387]), while diabetes mellitus was protective. The impact of TB persisted for six years after TB diagnosis and was significant in women and subjects aged >70 years. Among TB patients, delay in anti-TB treatment had a dose-response relationship with the risk of developing COPD.

**Conclusions:**

Some cases of COPD may be preventable by controlling the TB epidemic, early TB diagnosis, and prompt initiation of appropriate anti-TB treatment. Follow-up care and early intervention for COPD may be necessary for treated TB patients.

## Introduction

Chronic obstructive pulmonary disease (COPD), characterized by chronic airway inflammation with progressive lung function deterioration, is a major cause of morbidity, disability, and mortality [1]. It is the fourth leading cause of death worldwide, with a pooled prevalence of 6.4%, 1.8%, and 9.2% based on the definition of chronic bronchitis, emphysema, and airflow obstruction, respectively [2], [3]. Although tobacco smoking is recognized as an important risk factor of COPD, indoor air pollution like biomass fuel, occupational dusts, and exposure to certain gases, history of pulmonary tuberculosis (TB), chronic asthma, poor socio-economic status, and genetic factors are recognized as potential factors contributing to the development of COPD [4]. The prevalence of COPD among non-smokers is reported to be 6.6% and an estimated 25–45% of COPD patients have never smoked [4], [5]. A nationwide survey in South Africa suggests that in a TB endemic area, pulmonary TB may be the strongest risk factor of COPD [6].

Tuberculosis poses a global public health threat and remains the leading cause of death among infectious diseases, especially in undeveloped and developing countries [7]. While TB can occur in any organ or tissue, the respiratory system is the most common site of active disease. Without treatment, TB has characteristic chronic inflammation and has a mortality rate of 50% within five years [8]. Although standard anti-TB treatment is highly effective, with a rapid resolution of symptoms and low rate of relapse, non-adherence remains a great obstacle to successful treatment [9]. After completing treatment for pulmonary TB, about two-thirds of patients have pulmonary function abnormalities, with obstructive defect being the main abnormality [10]–[13].

Theoretically, delay or non-adherence in anti-TB treatment may increase the duration and severity of airway inflammation and lung destruction, thus rendering the development of COPD. However, the association between anti-TB treatment course and COPD has never been elucidated. This cohort study was conducted using the database of the National Health Insurance (NHI) of Taiwan to investigate the influence of pulmonary TB and delay and non-adherence of anti-TB treatment on the risk of developing COPD.

## Materials and Methods

The study has been approved by the institutional review board (IRB) of National Taiwan University Hospital, Taipei, Taiwan. As a retrospective study using an encrypted database, the IRB waved the need for informed consent for this study.

### Data Source and Case Selection

The NHI of Taiwan is a mandatory universal health insurance program offering comprehensive medical care coverage to all Taiwanese (Supporting Information S1). The Longitudinal Health Insurance Database (LHID) 2005, a subset database of the NHI program, contained the entire original claim data from 1996 to 2007 of 1,000,000 beneficiaries randomly sampled from the year 2005 Registry for Beneficiaries of the NHI program. From the LHID 2005, pulmonary TB cases (TB group) and control subjects (control group) matched in age, sex, and timing of entering the database were selected (1∶5 in case number).

### Definition of Active Tuberculosis

Active pulmonary TB was defined by at least two ambulatory visits or one in-patient record, with a compatible diagnosis plus at least one prescription consisting of three or more than three anti-TB drugs and prescriptions of at least two anti-TB drugs simultaneously for ≥120 days during a period of 180 days (Supporting Information S1). For patients with end-stage renal disease, the prescriptions were adjusted as advised in the treatment guidelines [14]. Patients with pulmonary TB onset prior to January 1, 1999 were excluded to ensure an observation period of three years to monitor active COPD treatment or diagnoses. Patients with <1 year of follow-up after pulmonary TB were also excluded because remaining period of observation was insufficient to confirm the subsequent development of COPD.

Because the health system delay in TB diagnosis had been reported to vary from 3 to 6 months [15], [16], the initial study date in the TB group was defined as the initial date of anti-TB treatment, and was moved forward if there were other respiratory diagnoses or orders of acid-fast smear, mycobacterial culture, bronchoscopy, pleural biopsy, or tuberculin skin test within 180 days prior to start of anti-TB treatment (Supporting Information S1). The delay in anti-TB treatment was defined as the interval between the initial study date and the date of the commencement of anti-TB treatment. In the control group, the initial study date of each subject was defined as the same day as the corresponding TB case.

### Definition of Chronic Obstructive Pulmonary Disease

The ambulatory care and in-patient discharge records were analyzed to recognize COPD patients. COPD was defined by at least two ambulatory or in-patient records with compatible diagnoses with prescription of at least two COPD-specific medications or one COPD-specific medication plus at least one airway medication(s) during a period of 90 days (Supporting Information S1). Patients with COPD onset prior to January 1, 1999 were excluded to ensure an observation period of three years to monitor the occurrence of active TB. In order to confirm the diagnosis of COPD, the total duration of active follow-up for COPD had to be >1 year and the confirmed date of COPD was therefore noted as 1 year later.

### Data Collection

Underlying co-morbidities and income status were also identified from the LHID 2005 (Supporting Information S1). The end study date was defined as the date COPD diagnosis was confirmed in COPD patients, and the end of follow-up in those without COPD. Subjects without COPD who canceled health insurance before December 31, 2007 were censored from survival analyses at the date of cancellation. The cumulative dosage of isoniazid, rifampicin, ethambutol, and pyrazinamide during the intensive phase (first two months) of anti-TB treatment was measured. Adherence to anti-TB treatment was estimated by number of days during the intensive phase covered by three or more anti-TB drugs and non-adherence by the number of days during the intensive phase covered by one or none of the anti-TB drugs.

### Statistical Analysis

Intergroup difference was calculated using independent-samples *t* test for continuous variables and *chi* square test or Fisher exact test for categorical variables, as appropriate. The degree of association between two variables was estimated by using Kendall correlation test. Time-to-event curves were generated using the Kaplan-Meier method and compared by log-rank test. Cox proportional hazards regression analysis was performed to evaluate the impact of age, gender, co-morbidities, income status, active pulmonary TB, and treatment-associated variables on risk of developing COPD (Supporting Information S1). The entry level and stay level in stepwise variable selection procedure were set at 0.15. Only variables with a two-sided *p*<0.05 were kept in final model. All analyses were performed using the SAS version 9.2 (SAS Institute Inc., Cary, NC, USA).

Since pulmonary function tests were not universally performed and their results were not available, the onset of COPD might precede its diagnosis by many years. Sensitivity analyses were performed, whereby the impact of pulmonary TB in different age groups and sub-populations with different follow-up durations were evaluated. Because tobacco use was uncommon in female Taiwanese (4.2%) [17], analysis was restricted to the sub-population of females to explore the impact of confounding bias by smoking.

## Results

**Figure 1:**
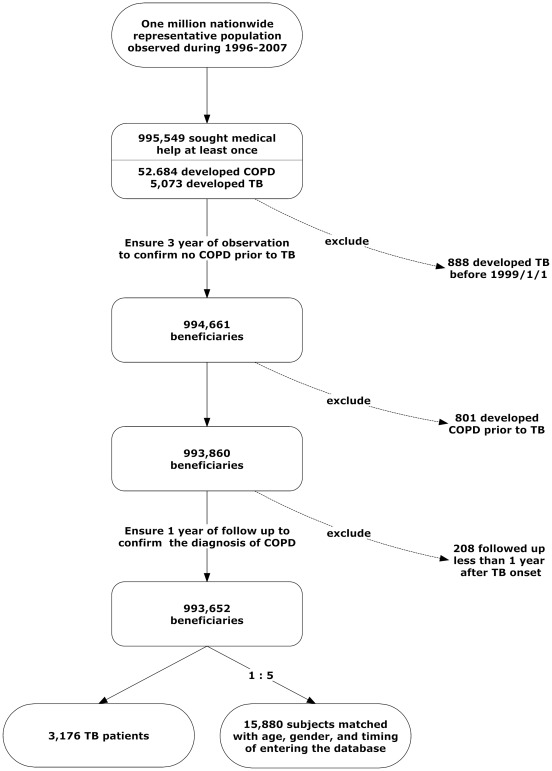
Case selection flow chart. The selection of pulmonary tuberculosis (TB) patients and age- and sex-matched control subjects from the Longitudinal Health Insurance Database 2005.

Among the 1,000,000 beneficiaries in LHID 2005, 995,549 had sought medical help at least once from 1996 to 2007. Among them, 52,684 and 5,073 subjects had COPD and pulmonary TB, respectively. A total of 3,176 cases fulfilled the diagnostic criteria of pulmonary TB and were selected as the TB group. Then 15,880 non-TB cases matched for age, gender, and timing of entering LHID 2005 were selected as the control group (Fig. 1). Their clinical characteristics assessed at the initial study date were summarized in Table 1. The mean age of TB cases were 51.9±19.2, with male predominance (male-to-female ratio, 1.9). The most common underlying co-morbidities in the TB group were diabetes mellitus and malignancy.

**Table 1:** Clinical characteristics of subjects at the initial study date.

	Tuberculosis, n = 3176	Control, n = 15880	*p* value
Age (year)	51.9±19.2	51.9±19.2	0.999
Male	2080 (65.5)	10400 (65.5)	1.000
Developing COPD	241 (7.6)	624 (3.9)	<0.001
Follow up duration (year)	4.4±2.3	4.5±2.2	0.005
in those developing COPD	2.3±1.4	2.5±1.3	0.050
in those not developing COPD	4.6±2.2	4.6±2.2	0.496
Cancelled health insurance	16 (0.5)	60 (0.4)	0.304
Diabetes mellitus	569 (17.9)	1292 (8.1)	<0.001
Malignancy	89 (2.8)	305 (1.9)	0.001
lung cancer	3 (0.1)	7 (0.0)	0.225
other cancer	86 (2.7)	298 (1.9)	0.002
End-stage renal disease	36 (1.1)	60 (0.4)	<0.001
Autoimmune disease	17 (0.5)	31 (0.2)	<0.001
Liver cirrhosis	6 (0.2)	13 (0.1)	0.114
Acquired immuno-deficiency syndrome	6 (0.2)	3 (0.0)	0.001
Organ transplantation	1 (0.0)	7 (0.0)	1.000
Low income	87 (2.7)	186 (1.2)	<0.001
Ever undergoing spirometry	103 (3.2)	381 (2.4)	0.006

Abbreviation: COPD, chronic obstructive pulmonary disease.

Data were either number (%) or mean ± SD.

Sixteen subjects (0.5%) in the TB group and 60 (0.4%) in the control group cancelled health insurance during follow-up. The interval between initial study date and commencement of anti-TB treatment (delay in anti-TB treatment) was 75.8±65.4 days. The TB group had significantly higher risk of developing COPD than the control group (7.6% vs. 3.9%, *p*<0.001 by *chi* square test). The onset of COPD was earlier in the TB group than in the control group (2.3 vs. 2.5 years, *p* = 0.050 by *t* test). Compared to non-TB subjects, the TB subjects were more likely to have underlying co-morbidities, including diabetes mellitus, malignancy, end-stage renal disease, autoimmune disease, liver cirrhosis, and acquired immuno-deficiency syndrome (AIDS).

**Figure 2:**
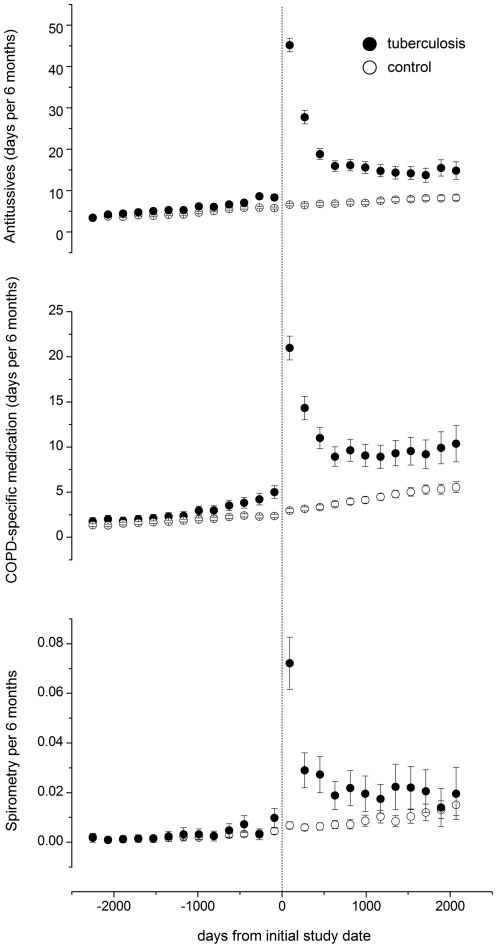
Prescription of antitussives, chronic obstructive pulmonary disease (COPD)-specific medication, and spirometry in tuberculosis patients and control subjects. Prescription data aggregated every 6 months from the individual initial study date and presented as mean (circle) and 95% confidence interval (error bar).

**Figure 3:**
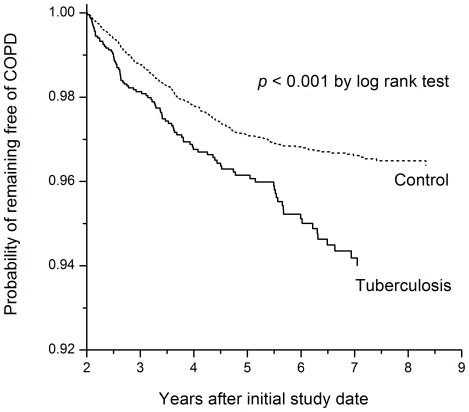
Curves of time to onset of chronic obstructive pulmonary disease (COPD) among tuberculosis and control groups.

Furthermore, more TB subjects had low income as compared to the control subjects. Comparing to controls, TB patients had more prescriptions of antitussives and COPD-specific medication after the initial study date, whereas the frequency of prescriptions was similar before the initial study date (Fig. 2). In addition, the proportion of TB patients who had undergone spirometry was higher before (3.2% vs. 2.4%, *p* = 0.006 by *chi* square test) and after (15.6% vs. 4.7%, *p*<0.001 by *chi* square test) the initial study date (Fig. 2). The time-to-COPD curves of the two groups were significantly different as calculated by log-rank test (Fig. 3).

Cox proportional hazards regression analysis, including all the variables listed in Table 1 for the 3,176 pulmonary TB cases and 15,880 control subjects, revealed that pulmonary TB was a risk factor for developing COPD (hazard ratio [HR] 2.054 [range, 1.768–2.387]) (Table 2). Age, male sex, and low income were risk factors for developing COPD, whereas diabetes mellitus was protective. Sensitivity analyses revealed that pulmonary TB remained an independent risk factor for developing COPD in cases of different follow-up durations after the initial study date, ranging from >2 to >6 years (Table 3) (Supporting Information S1). The TB HR (4.291 [range, 2.623–7.020]) for developing COPD among patients aged <40 years was higher than that among of patients aged >40 year (Table 3). However, pulmonary TB was an independent risk factor for developing COPD even among subjects older than 70 years. In the sensitivity analysis that included females only, pulmonary TB remained an independent risk factor for developing COPD, while the magnitude of association was higher than those of males or the overall population (HR 2.473 [range, 1.782–3.433]) (Table 3). Diabetes mellitus was not protective in the female sub-population.

**Table 2:** Independent risk factors for developing COPD in pulmonary tuberculosis patients (n = 3176) and control subjects (n = 15880), by Cox proportional hazards regression analysis.

	*p* value	Hazard ratio (95% CI)
Age	<0.001	1.047 (1.043–1.052)
Male	<0.001	2.001 (1.687–2.373)
Tuberculosis	<0.001	2.054 (1.768–2.387)
Diabetes mellitus	0.003	0.730 (0.591–0.902)
Low income	0.048	1.549 (1.004–2.390)

**Table 3:** Impact of pulmonary TB on developing COPD in sensitivity analyses.

Study population	*p* value	Hazard ratio (95% CI)
All subjects	<0.001	2.054 (1.768–2.387)
Cases of follow-up duration >2 years	<0.001	1.565 (1.249–1.960)
Cases of follow-up duration >3 years	0.003	1.577 (1.163–2.139)
Cases of follow-up duration >4 years	0.008	1.780 (1.160–2.731)
Cases of follow-up duration >5 years	<0.001	3.641 (2.024–6.550)
Cases of follow-up duration >6 years	0.008	3.361 (1.372–8.239)
Age ≤40 years	<0.001	4.291 (2.623–7.020)
Age >40 years	<0.001	1.937 (1.653–2.269)
Age >50 years	<0.001	1.836 (1.553–2.169)
Age >60 years	<0.001	1.684 (1.400–2.026)
Age >70 years	<0.001	1.607 (1.261–2.049)
Women	<0.001	2.473 (1.782–3.433)
Men	<0.001	1.975 (1.669–2.337)

Abbreviations: COPD, chronic obstructive pulmonary disease; TB, tuberculosis.

The clinical characteristics assessed at the initial study date of TB cases with or without developing COPD were shown in Table 4. The mean age of TB patients who developed COPD was 62.5±14.9 years, with male predominance (male-to-female ratio, 3.5). The most common underlying co-morbidity in TB cases developing COPD was diabetes mellitus, followed by malignancy. Among all TB cases, the median and mean delays in anti-TB treatment were 66 and 75.8 days, respectively. Delay in anti-TB treatment was ≤7, 8–90, and >90 days in 25.5%, 32.2%, 42.3% of TB cases, respectively. The entire treatment course strictly adhered to the TB treatment guidelines using the standard four-combined regimen (i.e., isoniazid, rifampicin, ethambutol, and pyrazinamide in the first two months, followed by isoniazid, rifampicin with or without ethambutol) in 71.3% of TB cases. Another 8.2% received the three-combined regimen (i.e., isoniazid, rifampicin plus ethambutol).

**Table 4:** Clinical characteristics at the initial study date of tuberculosis cases (n = 3176) with or without subsequent chronic obstructive pulmonary disease.

	Developing COPD, n = 241	Not developing COPD, n = 2935	*p* value
Age (year): mean	62.5±14.9	51.1±19.2	<0.001
Male	188 (78.0)	1892 (64.5)	<0.001
Diabetes mellitus	36 (14.9)	533 (18.2)	0.210
Malignancy	8 (3.3)	81 (2.8)	0.613
End-stage renal disease	0 (0.0)	36 (1.2)	0.108
Autoimmune disease	0 (0.0)	17 (0.6)	0.634
Liver cirrhosis	0 (0.0)	6 (0.2)	1.000
AIDS	0 (0.0)	6 (0.2)	1.000
Organ transplantation	0 (0.0)	1 (0.0)	1.000
Low income	9 (3.7)	78 (2.7)	0.325
Delay in ATT (days)	94.9±64.8	74.3±65.2	<0.001
No. of days in first 2 months of ATT			
receiving isoniazid	43.7±21.2	46.2±19.5	0.082
receiving rifampicin	48.2±13.0	49.9±12.0	0.041
receiving ethambutol	49.7±13.3	48.7±13.8	0.323
receiving pyrazinamide	45.0±21.2	48.1±19.8	0.031
without ATT	3.4±8.0	2.5±6.6	0.079
receiving ≤1 drug	6.0±9.7	5.2±9.0	0.194
receiving ≥3 drugs	47.7±13.1	49.4±12.4	0.044

Abbreviations: AIDS, acquired immuno-deficiency syndrome; ATT, anti-tuberculous treatment; COPD, chronic obstructive pulmonary disease.

Data were either number (%) or mean ± SD.

**Figure 4:**
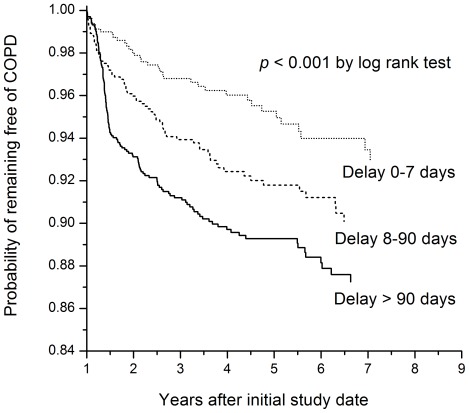
Curves of time to onset of chronic obstructive pulmonary disease (COPD) among TB patients with different delays in anti-tuberculous treatment.

Within the first two months of anti-TB treatment, the number of days receiving isoniazid had good correlation with the number of days receiving rifampin, pyrazinamide, ≥3 drugs, ≤1 drug, and without treatment (Kendall correlation coefficient: 0.344, 0.375, 0.573, −0.586, and −0.442, respectively). Compared to TB patients who did not develop COPD, those who developed COPD were older and were more likely to be male, and receive anti-TB treatment later with more interruption or modification in regimen. Kaplan-Meier analysis showed that a longer delay in anti-TB treatment was significantly associated with the development of COPD (Fig. 4).

Cox proportional hazards regression analysis was performed for the 3,176 pulmonary TB subjects to identify independent risk factors for developing COPD and showed that delay in anti-TB treatment was an independent risk factor (HR 1.005 [range, 1.003–1.007]) (Table 5). Age and male sex were risk factors for developing COPD, whereas diabetes mellitus was protective. In the sensitivity analysis of females only, delay in anti-TB treatment remained an independent risk factor and the magnitude of association was slightly higher (HR 1.009 [range, 1.004–1.013]). However, diabetes mellitus was not protective in the female sub-population.

**Table 5:** Independent risk factors for developing COPD in pulmonary tuberculosis (TB) patients (n = 3176), by Cox proportional hazards regression analysis.

	*p* value	Hazard ratio (95% CI)
Age	<0.001	1.036 (1.028–1.044)
Male	<0.001	1.812 (1.333–2.462)
Diabetes mellitus	0.003	0.587 (0.411–0.838)
Delay in anti-TB treatment (days)	<0.001	1.005 (1.003–1.007)

## Discussion

Using a national insurance database, the present study has two main findings. First, aside from the well-known predisposing factors of COPD like age, male sex, and low income, history of tuberculosis is an independent risk factor for developing COPD and its impact is sustained for at least six years after TB diagnosis. Second, among TB patients, delay in anti-TB treatment has a dose-response relationship with the risk of developing COPD. These findings have important clinical impact for the management of TB and COPD, suggesting that some COPD cases may be preventable by controlling the TB epidemic and improving the quality of TB diagnosis and treatment. Moreover, follow-up care and early intervention for COPD may be necessary for TB patients after anti-TB treatment.

Tuberculosis is characterized of chronic caseous granulomatous inflammation resulting in devastating tissue damage if left untreated [18]. As the most common site of involvement, pulmonary TB can cause permanent obstructive or restrictive pulmonary function impairment, which in turn can contribute to the pathogenesis of COPD [11]–[13]. In a nationwide survey in South Africa, the strongest predictor of COPD is a history of pulmonary TB, with odds ratio (OR) of 4.9 (95% CI 2.6–9.20) for males and 6.6 (95% CI 3.7–11.9) for females [6]. The study is, however, limited by the use of self-reported symptoms to define COPD.

By using post-bronchodilator pulmonary function (FEV1/FVC<0.70) to define COPD, subsequent case control studies supported the possible contribution of prior pulmonary TB history (OR 2.9–4.1) on COPD [19]. However, pulmonary TB was noted according to history and questionnaire only. The current analyses complement these reports by demonstrating the temporal association of TB preceding COPD at the population level using prospectively collected data. It is likely that the absolute effect is somewhat underestimated by the relatively insensitive definition of COPD. A previous study in patients with drug-susceptible pulmonary TB found that anti-tuberculous treatment can result in minimal structural and functional residua [20]. Consistently, the current findings suggest that a delay in initiating anti-TB treatment may further aggravate airway destruction and accelerate the development of COPD.

The initial presentation of TB is variable and sometimes non-specific, and easily attributed to other common clinical illness. Delay in initiation of treatment is appreciable for patients with either smear-positive or smear-negative pulmonary TB [21]. In addition to increasing the severity of TB and further transmission, delays in diagnosis and treatment may have long-term pulmonary sequelae and render the development of COPD. With the implementation of liquid mycobacterial culture system, the diagnosis of pulmonary TB for smear-negative disease still requires two weeks or more [22]. A high index of clinical suspicion and prompt mycobacteriologic studies, including acid-fast smears, mycobacterial culture, and even rapid molecular diagnostic test, are of practical importance for both TB and COPD.

Tobacco smoking is the most important risk factor for COPD [1]. It also increases susceptibility to TB and is associated with TB treatment failure [1], [17], [23]. Since a history of smoking and other environmental factors (e.g., biomass fuel exposure) are not available in the LHID 2005, it is possible that some of the effects of pulmonary TB on the development of COPD observed in present study are through the effect of toxic substances. In a previous report on COPD prevalence in 12 Asia-Pacific countries and regions [24], smoking and air pollution, rather than biomass fuel, were the most important risk factors of COPD in Taiwan. Men were more likely to have the two kinds of exposure than women. Results of sensitivity analysis in the present study that included females only reveals that even in this predominantly non-smoking population, previous TB and delay in anti-TB treatment are still independent risk factors for COPD. Though the sensitivity analysis cannot dissect the influences of these confounding factors, the current findings suggest that TB maybe a risk factor of COPD itself or a surrogate of inhalation of toxic substances and low socio-economic status.

In addition, Lee et al. recently demonstrated that prior history of TB was independently associated with airflow obstruction after adjusting for age, sex, and smoking status [25]. Radiologic severity of prior TB was also associated with the severity of non-smoker COPD [25]. All of these evidences emphasize that clinicians should keep a high index of suspicion for COPD in patients with prior TB history.

Another finding in the present study is that diabetes mellitus may have a protective effect on the development of COPD. This is probably because patients may stop smoking after diabetes mellitus is diagnosed, since the protective effect disappears when the analysis is restricted to the predominantly non-smoking female sub-population. The typical histologic finding of TB is caseous granulomatous inflammation, in which matrix metalloproteinases (MMPs) produced by monocytes and neutrophils are recently identified as key mediators for the matrix destruction [26]. The MMPs have also been shown to mediate the pathogenesis of COPD and predict its pulmonary function decline [27]. Meanwhile, diabetes mellitus is associated with monocyte and neutrophil dysfunction with impaired phagocytosis and migration [28], [29]. Although the finding has not yet been confirmed, it is possible that such immune cell dysfunction may modify matrix destruction and reduce the risk of developing COPD.

The present study has some limitations. First, mycobacteriologic results, pulmonary function test, and radiological findings were not available. Therefore, it is difficult to confirm the diagnosis of pulmonary TB and COPD. Other respiratory diseases, especially asthma, may be misclassified as COPD. However, the analysis reveals that pulmonary TB remains an independent risk factor even in subjects aged >70 years, a population that is more likely to have COPD rather than asthma. The conclusions here may be applied to the management of TB and COPD since the findings have been generated from the national insurance database, a record of medical practice in the real world.

Second, the prevalence of COPD may be underestimated, especially in the control subjects, because pulmonary function tests are less frequently prescribed in control subjects. However, in addition to spirometry, TB patients have more prescriptions of antitussives and COPD-specific medication than control subjects after the initial study date, suggesting that respiratory symptoms, a key feature of COPD, are more common during post-TB follow-up. Furthermore, the possibility of undiagnosed COPD prior to the initial study date may also still exist, despite an observation period of three years. Thus, the impact of TB increases as follow-up duration increased.

Third, without knowing the clinical reasons for the timing of treatment with specific anti-TB drugs, calculation of treatment delay is somewhat arbitrary and its impact on the development of COPD are probably less secure. Clinical studies with longer follow-up duration are warranted to fully understand the interaction between TB and COPD.

In conclusion, pulmonary tuberculosis is an independent and long-lasting risk factor for developing COPD. Any delay in initiating anti-TB treatment remains a serious issue and increases the risk of COPD. In order to control TB epidemics and reduce COPD burden, early diagnosis of pulmonary TB and prompt initiation of appropriate anti-TB treatment is imperative. Further studies on the post-treatment course of pulmonary TB are needed to optimize follow-up care and early intervention for COPD.

## Supporting Information

Supporting Information S1 Supplement file containing detailed information on study methodology and curves of time to onset of chronic obstructive pulmonary disease in cases of different follow-up durations.
(PDF)
