# Supplementary material for: Pulmonary Tuberculosis and Delay in Anti-Tuberculous Treatment Are Important Risk Factors for Chronic Obstructive Pulmonary Disease
Source: PLoS One. 2012 May 25;7(5):e37978. doi: 10.1371/journal.pone.0037978 (PMC3360660; doi:10.1371/journal.pone.0037978)
Supplement: Supporting Information S1 — Supplement file containing detailed information on study methodology and curves of time to onset of chronic obstructive pulmonary disease in cases of different follow-up durations. (PDF) [file pone.0037978.s001.pdf]

Online Supplement

METHODS

National Health Insurance of Taiwan

Up to 96% of the residents in Taiwan have been enrolled in the National Health Insurance (NHI) program since 1996 [1]. Comprehensive healthcare data includes enrollment files, claims data, catastrophic illness files, and the registry for drug prescriptions. All of the information in the Longitudinal Health Insurance Database 2005, a subset database of the NHI program that allows a specific individual patient to be identified, has been encrypted. The confidentiality of the data is guaranteed by the data regulations of the Bureau of National Health Insurance [2].

Definition of Active Pulmonary TB

The compatible diagnosis of active pulmonary TB included ICD-9-CM (International Classification of Diseases, 9th revision, clinical modification) code (010-012, 018) or A-code (A020, A021). The anti-tuberculous drugs used included isoniazid, ethambutol, rifampicin, pyrazinamide, protionamide, terizidone, streptomycin, kanamycin, quinolones, cycloserine, and aminosalicylic acid.

The initial study date for the TB group was defined as the initial date of anti-tuberculous treatment, and was moved forward if there were either of the following two conditions within 180 days prior to initiation of anti-TB treatment. First, there were diagnoses of cough (ICD-9-CM code 786.2), hemoptysis (ICD-9-CM code 786.3), sputum (ICD-9-CM code 786.4), lung nodule (ICD-9-CM code 786.6), acute airway infection (ICD-9-CM code 460, 464-466, A-code A320), pleural effusion (ICD-9-CM code 511, A-code A327),

pneumonia (ICD-9-CM code 480-487 or A-code A321), lung abscess (ICD-9-CM code 513), tuberculosis, pneumoconiosis (ICD-9-CM code 500-505 or A-code A326), bronchiectasis (ICD-9-CM code 494 or A-code A324), or pneumonitis (ICD-9-CM code 506-508). Second, there were orders for acid-fast smear, mycobacterial culture, bronchoscopy, pleural biopsy, or tuberculin skin test.

Definition of Chronic Obstructive Pulmonary Disease (COPD)

Compatible diagnosis of chronic obstructive pulmonary disease included ICD-9-CM codes 490-492, 496 or A-code A323, A325.

The COPD-specific medication was defined as prescriptions containing corticosteroids (inhaled, oral, or parenteral), beta-agonists (long-acting or short-acting; inhaled, oral, or parenteral), anti-cholinergics (i.e., ipratropium and tiotropium), aminophylline, or theophylline. Airway medication included oral antitussives, mucolytic agents, and sympathomimetics.

Co-morbidities and Income Status

Underlying co-morbidities were noted if they were present before the initial study date. Lung cancer was defined by a compatible ICD-9-CM code (162) or A-code (A101) from the Registry for Catastrophic Illness Patient Database (RCIPD), which is a separate file section of the NHI Database. Histologic confirmation is needed by the NHI of Taiwan to apply for this Catastrophic Illness Registry. Other co-morbidities were also investigated, including diabetes mellitus (ICD-9-CM code 250 or A-code A181 with prescription of insulin or oral hypoglycemic agents for >60 days), end-stage renal disease (ICD-9-CM code 585 or A-code A350 in RCIPD), liver cirrhosis (ICD-9-CM code 571 in RCIPD), acquired immuno-deficiency disease (five or more

records with compatible diagnoses [ICD-9-CM code 041, 044, V08 or A-code A049] and prescription of HAART medications), and autoimmune diseases (ICD-9-CM code 710, 714 or A-code A430, A431 in RCIPD). The low income group identified from the insurance status in the database generally had an annual household income <4500 US dollars [3].

Statistical Analysis

Basic model-fitting techniques, including variable selection, goodness-of-fit, area under the receiver operating characteristic (ROC) curve, adjusted generalized R^2, and regression diagnostics (i.e., residual analysis, detection of influential cases, and check for multi-collinearity) were applied to ensure the quality of multivariate analysis. Utilizing a stepwise variable selection procedure, all potential predictors were included.

REFERENCES

1. Bureau of National Health Insurance (2011) The National Health Insurance Statistics.
2. Wu CY, Hu HY, Pu CY, Huang N, Shen HC, et al. (2010) Pulmonary tuberculosis increases the risk of lung cancer: a population-based cohort study. Cancer.
3. (2010) Public Assistance Act. 7th ed. Taipei, Taiwan (R.O.C): Ministry of the Interior.

FIGURES

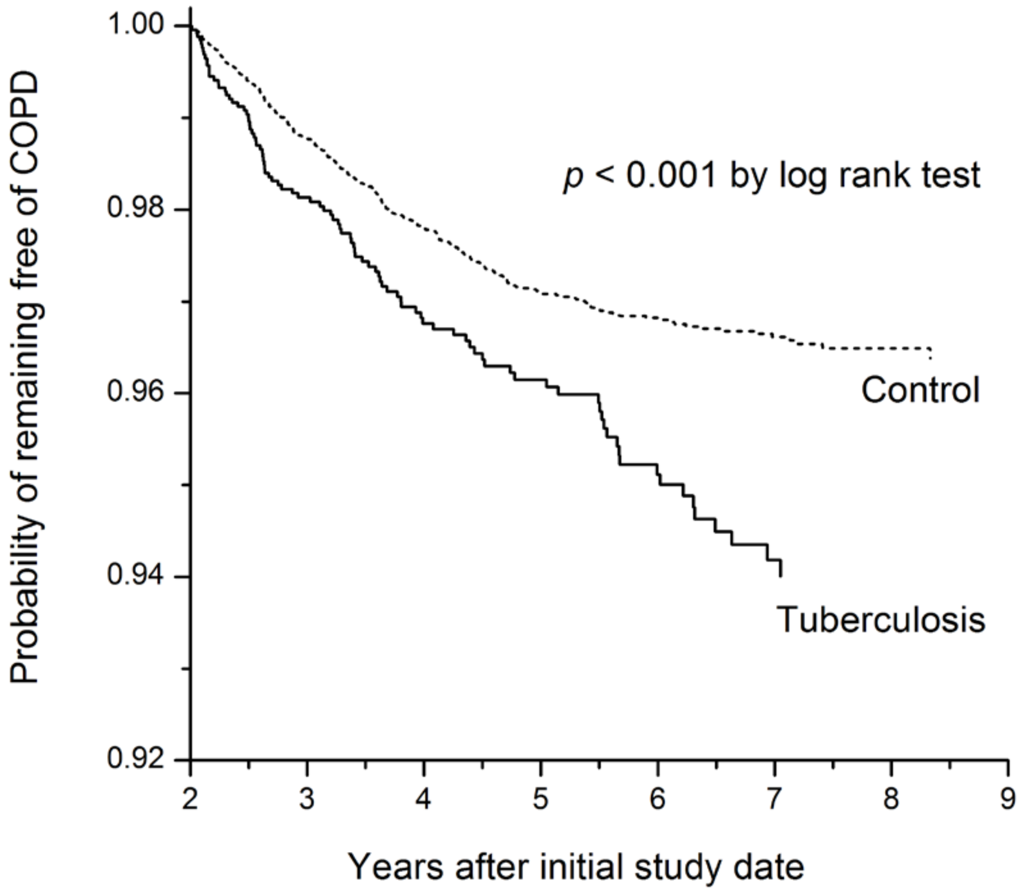

Figure E1. The curves of time to onset of chronic obstructive pulmonary disease (COPD) among tuberculosis and control groups, excluding cases with a follow-up duration ≤2 years after the initial study date.

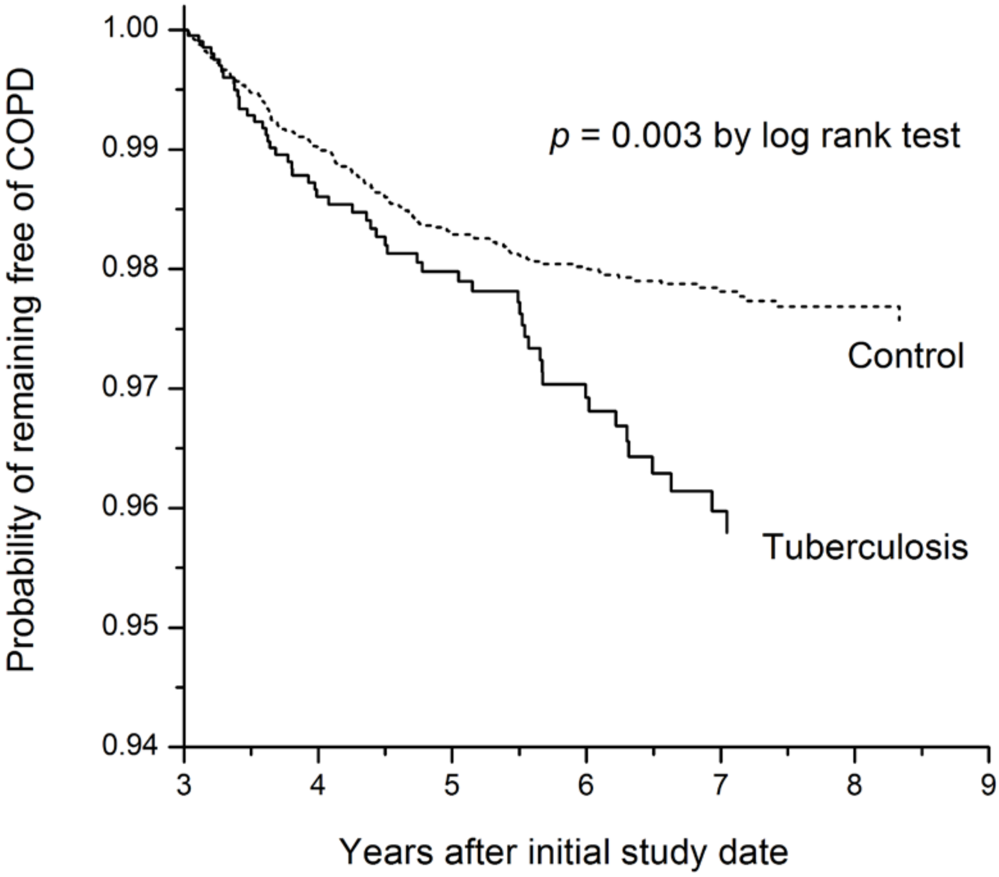

Figure E2. The curves of time to onset of chronic obstructive pulmonary disease (COPD) among tuberculosis and control groups, excluding cases with a follow-up duration ≤3 years after the initial study date.

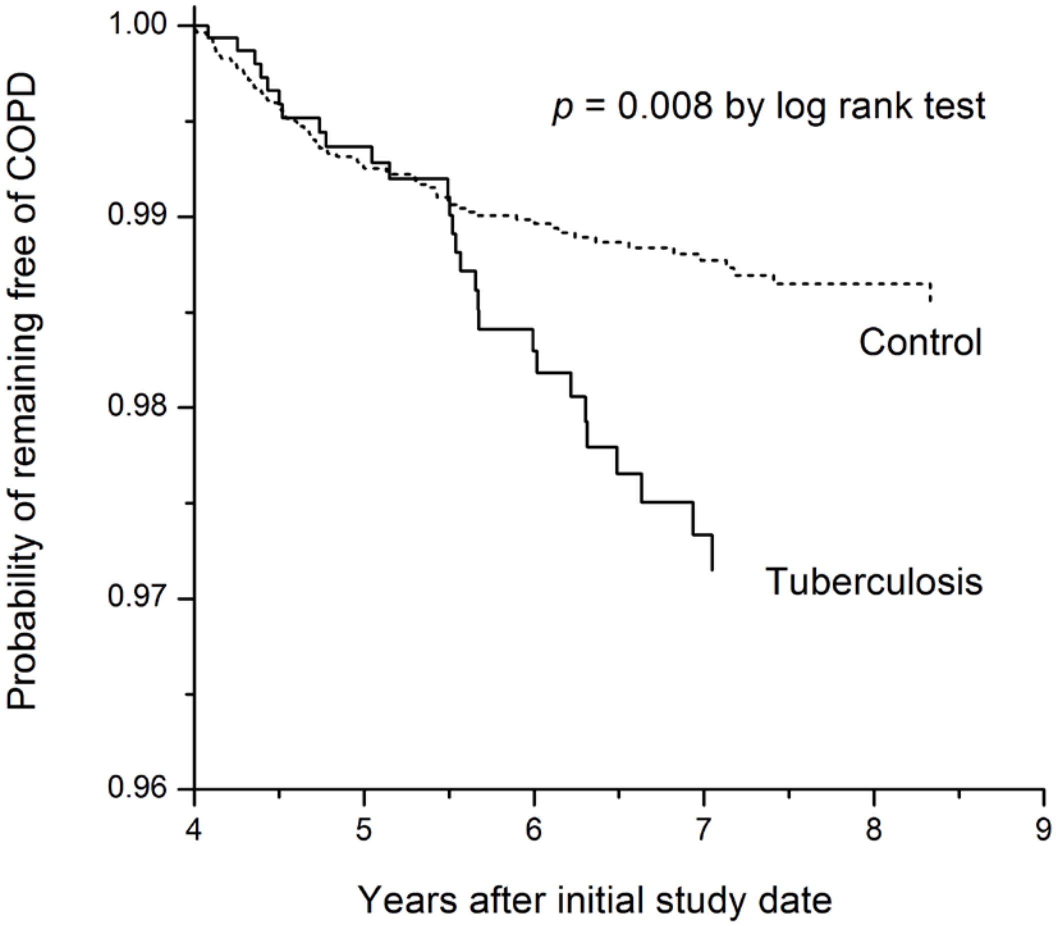

Figure E3. The curves of time to onset of chronic obstructive pulmonary disease (COPD) among tuberculosis and control groups, excluding cases with a follow-up duration ≤4 years after the initial study date.

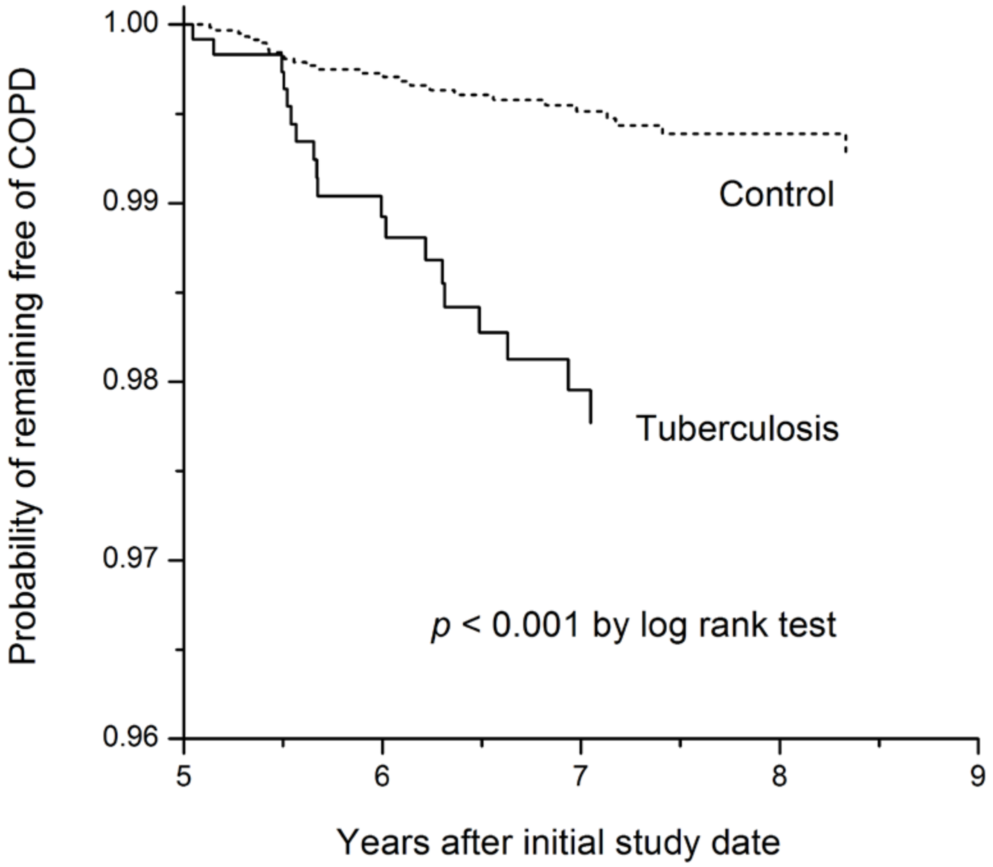

Figure E4. The curves of time to onset of chronic obstructive pulmonary disease (COPD) among tuberculosis and control groups, excluding cases with a follow-up duration ≤5 years after the initial study date.

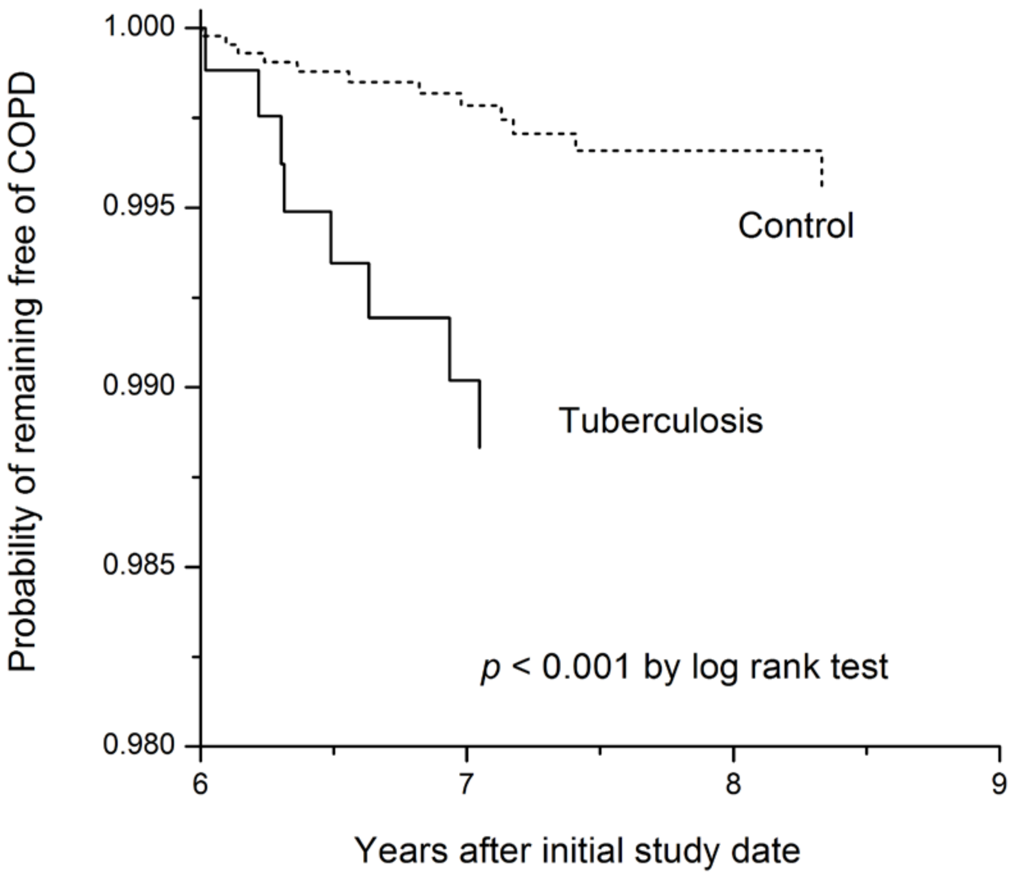

Figure E5. The curves of time to onset of chronic obstructive pulmonary disease (COPD) among tuberculosis and control groups, excluding cases with a follow-up duration ≤6 years after the initial study date.
